# Development of a conceptual framework of food and nutrition literacy in children

**DOI:** 10.1186/s40795-022-00590-z

**Published:** 2022-08-26

**Authors:** Azam Doustmohammadian, Nasrin Omidvar, Nastaran Keshavarz-Mohammadi, Hassan Eini-Zinab, Maryam Amini, Morteza Abdollahi

**Affiliations:** 1 Gastrointestinal and Liver Diseases Research Center, Iran University of Medical Sciences, Tehran, Iran; 2 Department of Community Nutrition, Faculty of Nutrition and Food Technology, and National Nutrition and Food Technology Research Institute, Shahid Beheshti University of Medical Sciences, Tehran, Iran; 3 School of Public Health and Safety, Shahid Beheshti University of Medical Sciences, Tehran, Iran; 4 Department of Nutrition Research, National Nutrition and Food Technology Research Institute, and Faculty of Nutrition and Food Technology, Shahid Beheshti University of Medical Sciences, Tehran, Iran

**Keywords:** Food literacy, Nutrition literacy, Children

## Abstract

**Background:**

This study aimed to develop a conceptual framework to better understand food and nutrition literacy and its dimensions and components among Iranian children.

**Methods:**

The study included three sub-studies. First, two qualitative studies were conducted to explore experts’ and students’ perspectives and experiences regarding the topic. A comprehensive qualitative literature review was then conducted to identify food/nutrition literacy definitions and its components in the relevant literature. The data of the above three sub-studies were compiled as text data and were analyzed utilizing MAXQDA_2010_ software.

**Results:**

Two main domains, including cognitive and skill domains, emerged from the data analysis. The cognitive domain consisted of food- and nutrition-related knowledge included four subcategories “nutrition knowledge”, “lifestyle knowledge”, “food safety knowledge”, and “knowledge of food and its preparation”) as well as “food and nutrition understanding”. The skill domain consisted of three dimensions: “functional”, “interactive”, and “critical” food and nutrition literacy.

**Conclusions:**

The developed framework highlights the importance of integrated application of all dimensions of food and nutrition literacy among this population group. It can assist policymakers and curriculum developers in assessing education curricula and developing effective strategies for teaching and learning to increase students’ food and nutrition literacy.

**Supplementary Information:**

The online version contains supplementary material available at 10.1186/s40795-022-00590-z.

## Background

Health Literacy is a fundamental skill to an individual’s ability to make appropriate health decisions [1]. Nutbeam provided a useful but general model of health literacy, describing functional, interactive, and critical literacy, which together create a progression of development of skills [2]. Using such insights, scholars have classified health literacy into different levels that progressively reflect individual empowerment in decision-making and involvement in both personal behaviors and social action to address the determinants of health [3, 4].

Nutrition literacy definition, which refers to Nutbeam’s tripartite model [2], can be defined as the capacity to access, understand, and apply nutritional information at three different levels, including functional, interactive, and critical nutrition literacy [4–6].

Food literacy which is more specific than nutrition literacy has been defined as the collection of interconnected information, skills, and behavior necessary to plan, manage, choose, prepare, and consume foods to fulfill needs and determine food intake [7]. Food literacy and nutrition literacy, often as separated terms, are extensively used in the policy, practice, research, and public arena [8]. However, there is no shared definition or understanding of its components or specific tools for different age groups, especially children [9].

Individuals’ ability to access health information and their motivation and skills to use information is greatly influenced by age, health status, and the context in which information might be applied [10], as well as the content of health information.

Recently, there have been some attempts to develop tools for measuring food and nutrition literacy among adults in Western societies [11–14], however data on food/nutrition literacy is limited for Asian countries [15, 16]. Furthermore, considering the cognition differences between adults and children, the above instruments cannot be used for assessing children.

Many dietary behaviors are established in childhood and can track through to adulthood [17]. Empowering elementary school children with food and nutrition literacy supports their self-efficacy regarding basic food preparation, healthy eating behaviors, and food choices [18]. Recently nutrition educators have emphasized the need for greater attention to developing food-related skills beyond teaching nutrition concepts alone. Evidence showed the potential benefits of integrating nutrition education curriculum with skill-building programs in schools to foster healthy eating behaviors in children [19, 20].”

Childhood provides an opportunity for health practitioners to promote the adoption of healthy behavior and prevent the development of health problems later in adulthood [21–23]. However, there is no well-developed and commonly agreed on conceptual framework and tools for food and nutrition literacy to be used by health researchers and practitioners to develop effective interventions for children. Our study aims to address these gaps within a varied sample of school-age children. This study aims to 1) identify dimensions and components of food and nutrition literacy and 2) develop a conceptual framework for food and nutrition literacy in Iranian children.

## Methods

### Study design

We carried out a qualitative study utilizing focus groups (FGs) and in-depth interviews as well as a comprehensive literature review to identify the dimensions and main components of food and nutrition literacy in children. Following the Standards for Reporting Qualitative Research (SRQR) [24] and the Consolidated Criteria for Reporting Qualitative Research (COREQ) the materials and methodology of the study will be provided [25].

### Procedure

Using Nutbeam’s hierarchy of health literacy as a guide [2], the study was designed in three sub-studies, including 1) The study of food and education experts to explore their views of food and nutrition literacy concept and its components in children, 2) The study of primary school students to identify nutrition knowledge, skills and behavior of children required to meet their food and nutrition needs and 3) A comprehensive literature review to explore available food literacy/nutrition literacy definitions and its components in current literature.

**Fig. 1:**
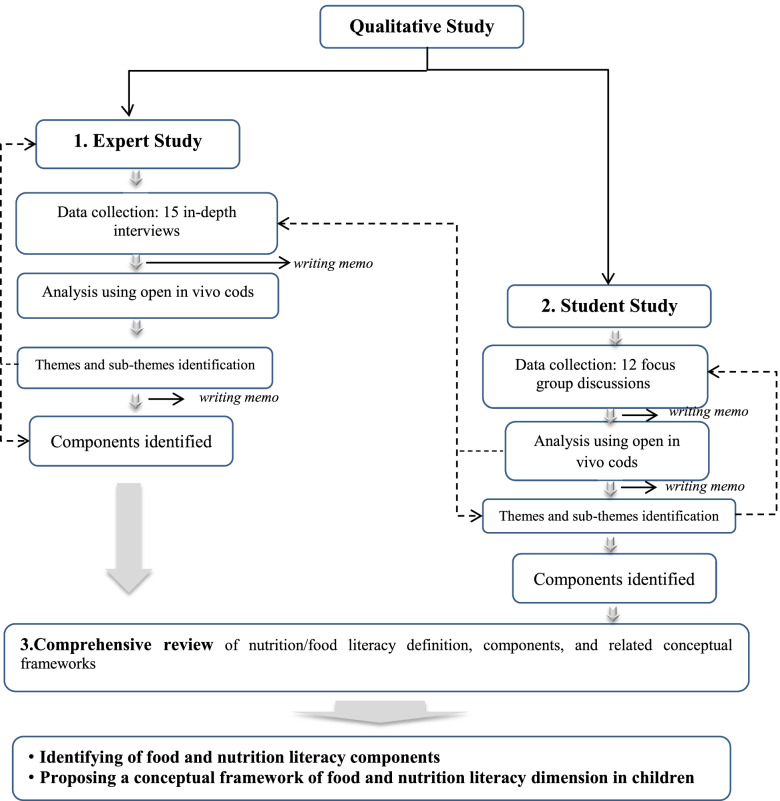
Research design

An overview of the study’s design is shown in Figure 1. The results of three sub-studies were merged at the end to provide a comprehensive understanding and perspective to extract food and nutrition literacy dimensions and its components.

1. The study of food and education experts-Participants were selected through snowball and purposive sampling technique, with maximum diversity. The main researcher conducted interviews and principal supervisor using semi-structured questions (see additional file 1 for details). Two experts reviewed the content of the questions, and based on their feedback, minor changes were implemented. The food and education experts were selected based on the following criteria: 1) experts with enough food and nutrition-related experience, health education, and primary education; 2) representatives of the Iranian nutrition society. A snowball sampling strategy was utilized to draw additional expert members and account for the potential implicit bias of investigator selection. Among 18 contacted people, fifteen in-depth interviews were conducted, including eight nutritionists, three health education and promotion specialists, three public health specialists, and one teacher (response rate 83.3%). The duration of each session ranged between 30 to 80 min. Interviews were recorded and transcribed.
2. The study of primary school students-Twelve focus group discussions (FGDs) with an average of 7 participants per group and a maximum of 11 were conducted. In order to maximize the heterogeneity of the sample, two schools from each of the three different socio-economic areas of Tehran Metropolitan, including district 2 (affluent area), district 9 (semi-affluent area), and district 19 (deprived area), were included. Eighty-nine students aged 10 -12 years in fifth and sixth-grade were randomly selected from these schools to participate. The sample was varied in terms of gender, grade, and level of socio-economic status (Table 1 details demographic characteristics of the student participants). The main researcher performed all of the focus groups with assistance from an assistant moderator. Focus group discussion lasted between 1 to 2 h and was conducted in a private room using a semi-structured guide (see additional file 1) consisting of open-ended, probing questions, enabling respondents to fully explain their personal opinions, perceptions, and experiences.
3. A comprehensive review was conducted concurrently with the student study. Scholarly electronic databases, including PubMed, ISI, Science direct, Scopus, and Google Scholar using the following keywords, including “food literacy”, “nutrition literacy”, and “food skill” were searched. After the first search, the second search was undertaken to cross-reference results from the original search, and finally, a citation check was undertaken. Inclusion criteria were based on the contribution of the study to the science of food literacy/nutrition literacy. For example, it needed to add to the literature by conceptualizing the term (definition or discussion of components). Hence, studies that simply mentioned the term “food literacy/nutrition literacy” were not included for data extraction.

**Table 1:** Demographic characteristics of the student participants

**students participated in Focus Group Discussion** **(*n* = 89, age range 10–12 years, M ± SD = 11.07 ± 0.57 years)**						
**Sex/Grade**	**Girls (*n* = 42)**			**Boys (*n* = 47)**		
	**Grade 5**^**th**^** (*n* = 21)**	**Grade 6**^**th**^** (*n* = 21)**	**Total**	**Grade 5**^**th**^** (*n* = 22)**	**Grade 6**^**th**^** (*n* = 25)**	**Total**
	N (%)	N (%)	N (%)	N (%)	N (%)	N (%)
**Area**						
**Affluent (district 2)**	7 (33.3)	7 (33.3)	14 (33.3)	7 (31.8)	11 (44)	18 (38.3)
**semi-affluent (district 9)**	7 (33.3)	7 (33.3)	14 (33.3)	8 (36.4)	7 (28)	15 (31.9)
**deprived (district 19)**	7 (33.3)	7 (33.3)	14(33.3)	7 (31.8)	7 (28)	14 (29.8)

### Ethics statement

The National Nutrition and Food Technology Research Institute’s ethics committee, Shahid Beheshti, University of Medical Sciences, approved the study (No.1394.20, 16-10-2015). Participants and the children’s parents provided written informed consent before the beginning of interviews and focus group discussions, and explicit consent was obtained before audiotaping.

### Data analysis

Transcripts of all fifteen interviews and twelve focus groups were imported into MAXQDA, 2010. For data analysis, directed thematic analysis was guided by Nutbeam’s hierarchical model of health literacy [2]. Interviewing was stopped when theoretical saturation was reached.

The Credibility and conformability of the data were established by prolonged, in-depth engagement with participants and member checking. Selecting participants with a range of experiences raises the chance of providing insight into the research issue from various aspects [26]. Two independent coders undertook to code, and inter-rater reliability was calculated using percent agreement (inter-rater reliability *r* = 0.90) [27]. This agreement indicated the reliability of the coding lists. Some of the participants contributed to check the results to determine if the codes were in line with their views. For the objective of transferability, all research-related specifics, including including procedures, actions, and decisions, were documented.

## Result

Data analysis of both studies (expert and student study) was merged into the themes developed at the first study. In both expert and student studies, food and nutrition literacy were found to be highly contextual. The final analysis of data from three sub-studies led to identifying thirteen components, and two main domains of cognitive domain and skill domain are presented in Table 2. More explanations are presented as follows.

**Table 2:** Food and nutrition literacy dimensions and its components in children

**Domains**	**Dimensions/Constructs**	**Components**
**Cognitive**	Food and nutrition related knowledge	i) Nutrition knowledge a. Knowing food groups, their nutritional values and the role of macronutrients b. Knowing food guidelines, balance, diverse and moderate diet c. Knowing the importance of main meal and snacks d. Knowing healthy and unhealthy foods e. Knowing the sources of nutritional information f. Understanding the acquired nutritional information
		ii) Lifestyle knowledge a. Knowing the interaction between food intake and physical activity b. Knowing relationship between common non-communicable (e.g. obesity and overweight, …) diseases and dietary pattern
		iii) Food safety knowledge a. Knowing basic personal health b. Knowing the preparation of foods with healthy materials c. Recognizing safe places to buy foods d. Knowing the basics of food storage (in stores, after purchasing…) e. Understanding the importance production and expired date f. Recognizing of signs of food contamination and unhealthy packaging
		iv) Knowledge of food and its preparation a. Knowing the food chain and its processing b. Cooking and food preparation knowledge
	Understanding	i)Understanding food and nutrition information
**Skills**	Functional	i) Access a) Ability to search and obtain the required food and nutrition information from reliable sources
		ii) Applying a. Healthy eating behavior and health - Drawing up and feeding behavior - Ability to prepare healthy snacks - Having activity - Personal hygiene b. Food choices - Purchasing based on production and expire date and standard sign - Choose healthy foods (e.g. healthy packed and stored in a suitable place) - Choose foods based on food labeling - Choose healthy foods and eating from a buffet / shop
	Interactive	i) Interactive skills a. Communicate with parents in order to provide healthy eating pattern b. Ability to exchange food and nutrition information with others
		ii) Emotional skills a. The ability to resist unhealthy food cravings and desires b. Saying ‘no’ skill to the temptation of unhealthy eating
		iii) Discussion Skills a. The ability to reason for opposition to other unhealthy eating behavior
	Critical	i) Media literacy a. Ability to analyze advertising and nutritional claims in the mass media
		ii) Food label literacy a. Ability to interpret nutritional information on labels and nutritional markers
		iii) Decision-making and planning a. Manage food choices are limited in terms of selection b. Economic management to buy healthy food c. Ability to plan for their health, family and surroundings

### Cognitive domain

#### Food and nutrition-related knowledge

In the cognitive domain, food and -related knowledge was the first concept that developed during continuous analysis and data comparisons. This concept represents the food and nutrition-related basic knowledge, which was essential for children based on the experts’ view. This concept encompasses four components: food and nutrition knowledge, lifestyle knowledge, food safety knowledge, and the knowledge of food and food preparation. One of the nutrition experts of UNICEF described: *“The students of 5*^*th*^* and 6*^*th*^* grade are expected to be aware and understand basic food and nutrition information to make decisions to improve their nutritional and know how proper diet and nutrition play a role in preventing obesity.”*

Most experts considered that having an overall concept of nutrition basics and the balance of foods was more important than more detailed information for this age group. A nutrition expert of Ministry of Health and Medical Education argued: *“In my opinion, a student of 5*^*th*^* and 6*^*th*^* grade should be aware of nutrition basics, that is he/she should know about food groups and their nutrition value, and he/she should be familiar with varied, well-balanced diet, the students should know what meals are and why and how they should eat meals; what are healthy snacks and why they should eat them….*”.

#### Understanding food and nutrition information

Most experts believe that food and nutrition literacy means that students have the capacity to apply food and nutrition information to the health situation, which requires understanding and interpreting the often-complex information about food and nutrition.

According to students’ views, lack of food and nutrition information in plain and understandable language was a significant barrier to using this information. “*Sometimes I don’t understand the nutrition information so, I ask from my teacher but sometimes he cannot explain things to me in plain or even in terms I know.”(A student in grade 5).*

### Skill domain

#### Functional food and nutrition literacy

##### Access

According to experts’ views, the ability to seek and identify reliable sources of nutritional information is one of the key required skills. Interviews indicated that most children asked their questions about food and nutrition information from parents, teachers and searched on the internet.

##### Applying

Nutritionists stated that in addition to the ability to seek required food and nutrition information, students should apply this information in their lifelong eating behaviors. This concept can be considered both a component and a potential outcome of food and nutrition literacy using both studies. In relation to food choice skills, one of the students said, “*when I want to buy something, I check production and expiration date and standard sign. I also check its ingredients*”. (A student in grade 5)

Some of the students mentioned their skills in preparation some food such as; “*Scrambled egg, Macaroni, Omelette and Cutlet….*” (A student in grade 6).

#### Interactive food and nutrition literacy

##### Interactive skills

According to experts’ views, enhanced confidence contributed to new ways of communicating about food and nutrition issues with peers, friends, and children, highlighting the social aspects of food and nutrition literacy. A nutritionist from a research site stated that*” one should share food and nutrition information with others because in interactive literacy we would like individuals to apply the food and nutrition information in their relationships. This relation may be with a nutritionist, teacher, parents, and peer and…”*

##### Emotional skill

The ability to withstand cravings and non-nutritious foods and the skill of saying “No” to unhealthy eating temptations that two of the nutritionists in the Ministry of Health and one of the nutritionists from the education system referred to conceptualized as emotional skill included in interactive food and nutrition literacy skills.

##### Discussion skills

Among the participants’ statements, the ability to argue and disagree with the non-nutritional behavior of peers and the family was one of the skills that were included in this domain.

*“One of the other skills is that if the family were unhealthy in their food behavior, then the person could convince them or be able to independently manage own diet.”* (A nutritionist from education system)

#### Critical food and nutrition literacy

The main identified components in this dimension were media literacy, food label literacy, and decision-making and planning skill.

##### Media literacy

One of the concepts in the process of continuous data analysis was media literacy. Today, we live in an advertisement-saturated world where consumption of unhealthy food and fostering unhealthy eating habits are promoted. Children as loyal and persistent customers of food marketing are exposed to and deceived by these misleading advertisements. According to experts in our study, developing media literacy in children is an essential skill that empowers them to evaluate and respond to media advertisements. In our study, most students did not trust the advertisements, and some of them believed they should investigate the trustworthiness of the advertisements. A student in grade 5 said: *“Advertisements are not true, and most of them are wrong and sell their products. I saw a fruit juice advertisement saying that fruit juice is made from real juice, but when I bought it and read its ingredients, it consisted of water, sugar, and flavors.”*

##### Food label literacy

In the expert study, participants referred to the importance of food labeling. They believed it was one of the best ways to transmit nutritional messages at the point of purchase. One of the nutritionists in the Ministry of Health, while pointing out the initiation of mandatory traffic lights, stated that *“traffic lights are the first and most important guide in which the consumer can communicate with food products through color markers. Traffic light may be more practical for children because of their simplicity.”*

##### Decision-making and planning skills in special condition

Managing money and time for preparing healthy food, healthy decision making in particular circumstances, and the ability to plan for own health, others, and the surrounding environment, were the components referred by participants. This component helps the individual minimize the impact of restricted resources or other special situations on healthy food choices.

A total of twenty-nine studies were included in the final review. Of these, 21 studies simultaneously addressed both food/nutrition literacy definitions and its components [5–8, 28–44], three studies only defined food/nutrition literacy [45–47] and 8 focused on food/nutrition literacy conceptual framework and its components [7, 8, 30, 31, 36, 38, 47, 48] in youth and adults. The main elements of all conceptual frameworks of food literacy included nutrition basic knowledge, and skills required to regulate food intake, planning meals, selecting and preparing food. Only three conceptual frameworks of food literacy comprised skills such as sharing information and interacting with others [7, 36, 38, 39]. All definitions of nutrition literacy and half of the food literacy definitions were based on an existing definition of health literacy [6, 7, 31, 36, 38]. The most emphasized components in “food literacy” were food skills including management, food preparation, and food selection as well as critical literacy [7, 38, 49, 50]. Slater (2013) presented food literacy using Nutbeam’s idea of health literacy as a framework for the food and nutrition curriculum of schools [6]. Based on the literature, all definitions of nutrition literacy focused on individuals’ cognitive abilities and strongly emphasized fundamental literacy and numeracy abilities required to comprehend and utilise nutrition-related information (functional skills). However, none provided a conceptual framework for nutrition literacy. For a general overview, see additional file 2.

### Conceptual framework of food and nutrition literacy in children

**Fig. 2:**
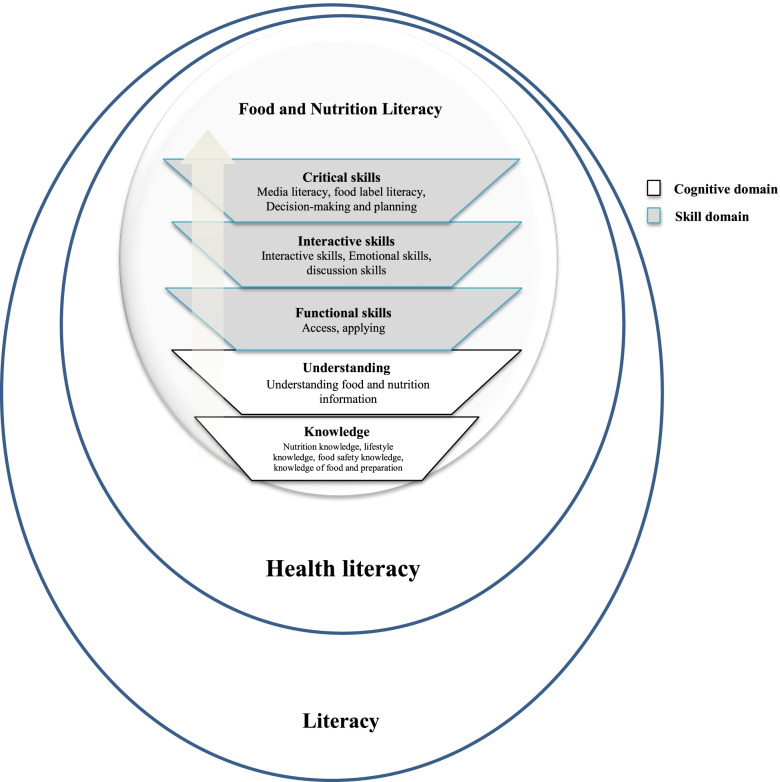
Food and Nutrition Literacy conceptual framework in children

Figure 2 shows the final framework developed in two domains of knowledge and skill but five dimensions. Each dimension included various components presented in Table 2.

## Discussion

This study proposed a comprehensive food and nutrition literacy model as a specific form of health literacy in children’s context. It is characterized by five dimensions: knowledge and understanding in the cognitive domain and functional, interactive, critical in skill domain. Findings indicated that food and nutrition literacy goes beyond having basic knowledge about food and nutrition. Food and nutrition literacy is not only the ability to use food and nutrition concepts and information but also the ability to interact nutritional information with others (peers, family, and nutritionists) and participate in social issues to promote community healthy eating patterns.

Although some definitions and conceptual models of food/nutrition literacy are documented, none of them are comprehensive enough to cover all evolving food/nutrition literacy definitions, with the competencies they imply for elementary school children. The current study helps fill this gap in three different ways. First, in the conceptual framework developed in this study, emotional and discussion skills, as well as food label literacy as the critical literacy components, were included. These remained the least captured in previous frameworks and tools [6, 7, 29, 36, 38, 39] while they are essential skills in the early years of life with great impact on lifelong health outcomes [51]. Children who develop discussion and emotional skills find it easier to manage their choices, resolve disputes about their conflicting priorities, share dietary information with others, and take part in initiatives to remove obstacles [52]. Second, an integrated concept of “food and nutrition literacy” was applied. Most experts believed there was no border between nutrition and food literacy in the current qualitative study. Findings from a recent systematic review have also shown that two concepts of food literacy and nutrition literacy have significant overlaps and can complete each other [53]. Third, it takes a dynamic systems approach to the components of food and nutrition literacy, showing that they are linked with each other to influence each other. Hence, it locates them in a multi-layer structure of health literacy as a whole.

According to the current conceptual framework, food and nutrition literacy encompasses the knowledge and skills that students need to access, understand, interpret, express ideas and opinions, interact (food and nutrition) information with others (peers, family, and nutritionists), analyze and evaluate food and nutrition information, and participate in activities related to health and nutrition in and out of schools. Success in any area means to be able to use the significant, identifiable, and distinctive food and nutrition literacy that is important for learning and representative of the content of that area. Nevertheless, food and nutrition knowledge can have a key role in fostering healthy eating behavior, while food skills are needed for translating knowledge into practice [54]. Developing skills typically provides some knowledge because practicing skills leads to acquiring knowledge [41, 54]. This viewpoint offers an expanded conceptualization of food and nutrition literacy beyond the acquisition of nutritional knowledge, which can lead to the empowerment of the community [28, 54].

It should be noted that all components may not always be present in every individual. Conversely, when a component is missing, the relationship with food and nutrition will be weaker and less able to respond to change in that area. It is important to be taken into account in developing food and nutrition literacy interventions.

We believe that good food and nutrition literacy could empower children and adolescents –who are especially vulnerable and, to some extent, are marginalized social groups –to become more engaged with the quality of their nutrition and more informed and reflective upon its status [41]. The linkage between nutrition-related knowledge, skills, and critical decision making, which are emphasized in our food and nutrition literacy framework, lead to enabling children to control their eating behavior and nutritional health determinants through involvement in food preparation, interpretation of claims made in food marketing and advertising and healthier food choices [7]. The development of food and nutrition skills can be enhanced if children have prior food and nutrition knowledge [54].

The developed framework in our study provides a common language and a vehicle for discussing the definition of the food and nutrition skills area in children. Such a discussion allows us to build consensus around the framework and measurement goals (Kirsch, 2001). Our current study was focused on school-aged children. While a valuable insight was gained into this group, the suggested conceptual framework is also transferrable across similar contextual and age-group of other Asian countries. This view is critical when considering the development of food and nutrition literacy instruments and interventions that aim the improvement functional, interactive, and critical skills in children.

### Limitation

To our knowledge, this study is the initial effort to propose a comprehensive food and nutrition conceptual framework for children. It is noteworthy that in the qualitative study, the true data saturation was difficult to be achieved; however, we supposed that the purposeful sampling for a larger sample besides the homogeneity of the samples might help overcome the shortcoming.

## Conclusion

This study highlights the importance of integrated application of all dimensions of food and nutrition literacy among this population group. It provides a framework for food and nutrition literacy and its components for elementary school children in Iran, which can be used by other countries and in developing tools to measure food and nutrition-related literacy and interventions for its improvement.

The findings also provide policymakers and curriculum developers a framework for evaluating educational curriculum and creating useful teaching and learning methods to increase students’ food and nutrition literacy.

## Supplementary Information

**Additional file 1: Table S1.** Question guides.

**Additional file 2: Table S2.** Food/nutrition literacy definitions and conceptual frameworks with components of functional, interactive and critical food/nutrition literacy.

## Data Availability

Data will be made available upon request from the corresponding author.

## Abbreviations

FNLIT: Food and nutrition literacy
NCDs: Non-communicable diseases
BMI: Body mass index
UFNI: Understanding Food and Nutrition Information
NHK: Nutritional health knowledge
FFNL: Functional food and nutrition literacy
IFNL: Interactive food and nutrition literacy
FCL: Food choice literacy
CFNL: Critical food and nutrition literacy
FLL: Food label literacy

## References

[1] Smith BJ, Tang KC, Nutbeam D (2006). WHO health promotion glossary: new terms. Health Promot Int.

[2] Nutbeam D (2008). The evolving concept of health literacy. Soc Sci Med.

[3] Nutbeam D, Kickbusch I (2000). Advancing health literacy: a global challenge for the 21st century. Health Promot Int.

[4] Pettersen S, Kjøllesdal JG, Aarnes SB (2009). Measuring nutrition literacy. Paper presented at the 19th International Conference of Nutrition, Bangkok, Thailand.

[5] Silk KJ, Sherry J, Winn B, Keesecker N, Horodynski MA, Sayir A (2008). Increasing nutrition literacy: testing the effectiveness of print, web site, and game modalities. J Nutr Educ Behav.

[6] Slater J (2013). Is cooking dead? The state of home economics food and nutrition education in a Canadian province. Int J Consum Stud.

[7] Vidgen HA, Gallegos D (2014). Defining food literacy and its components. Appetite.

[8] Bublitz MG, Hansen J, Peracchio LA, Tussler S (2019). Hunger and Food Well-Being: Advancing Research and Practice. J Public Policy Mark.

[9] Chung LMY (2017). Food Literacy of Adolescents as a Predictor of Their Healthy Eating and Dietary Quality. J Child Adolesc Behav.

[10] Velardo S (2015). The Nuances of Health Literacy, Nutrition Literacy, and Food Literacy. J Nutr Educ Behav.

[11] Carbone ET, Zoellner JM (2012). Nutrition and health literacy: a systematic review to inform nutrition research and practice. J Acad Nutr Diet.

[12] Poelman MP, Dijkstra SC, Sponselee H, Kamphuis C, Battjes-Fries MC, Gillebaart M, Seidell JC (2018). Towards the measurement of food literacy with respect to healthy eating: The development and validation of the self perceived food literacy scale among an adult sample in the Netherlands. Int J Behav Nutr Phys Act.

[13] Amin SA, Panzarella C, Lehnerd M, Cash SB, Economos CD, Sacheck JM (2018). Identifying food literacy educational opportunities for youth. Health Educ Behav.

[14] Park D, Park YK, Park CY, Choi M-K, Shin M-J (2020). Development of a comprehensive food literacy measurement tool integrating the food system and sustainability. Nutrients.

[15] Ashoori M, Omidvar N, Eini-Zinab H, Shakibazadeh E, Doustmohamadian A (2020). Development and validation of food and nutrition literacy assessment tool for Iranian high-school graduates and youth. Int J Prev Med.

[16] Durmus H, Gökler ME, Havlioğlu S (2019). Reliability and validity of the Turkish version of the short food literacy questionnaire among university students. Prog Nutr.

[17] Brown JE (2016). Nutrition through the life cycle: Cengage Learning.

[18] Cunningham-Sabo L, Lohse B (2013). Cooking with Kids positively affects fourth graders’ vegetable preferences and attitudes and self-efficacy for food and cooking. Child Obes.

[19] Savoie-Roskos MR, Wengreen H, Durward C (2017). Increasing fruit and vegetable intake among children and youth through gardening-based interventions: a systematic review. J Acad Nutr Diet.

[20] Hersch D, Perdue L, Ambroz T, Boucher JL (2014). The impact of cooking classes on food-related preferences, attitudes, and behaviors of school-aged children: a systematic review of the evidence, 2003–2014. Prev Chronic Dis.

[21] Birch LL, Savage JS, Fisher JO (2015). Right sizing prevention. Food portion size effects on children’s eating and weight. Appetite.

[22] Mikkila V, Rasanen L, Raitakari OT, Pietinen P, Viikari J (2004). Longitudinal changes in diet from childhood into adulthood with respect to risk of cardiovascular diseases: The Cardiovascular Risk in Young Finns Study. Eur J Clin Nutr.

[23] Organisation WH: Strengthening the health sector responses to adolescent health and development; 2010. [http://www.who.int/child_adolescent_health/documents/cah_adh_flyer_2010_12_en.pdf] Available from: [Accessed 20 Jan 2021].

[24] O’Brien BC, Harris IB, Beckman TJ, Reed DA, Cook DA (2014). Standards for Reporting Qualitative Research: A Synthesis of Recommendations. Acad Med.

[25] Tong A, Sainsbury P, Craig J (2007). Consolidated criteria for reporting qualitative research (COREQ): a 32-item checklist for interviews and focus groups. Int J Qual Health Care.

[26] Denzin NK, Lincoln YS (2009). Qualitative research.

[27] Rourke L, Anderson T, Garrison DR, Archer W (2001). Methodological issues in the content analysis of computer conference transcripts. Int J Artif Intell Educ.

[28] Zoellner J, Connell C, Bounds W, Crook L, Yadrick K (2009). Nutrition literacy status and preferred nutrition communication channels among adults in the Lower Mississippi Delta. Prev Chronic Dis.

[29] Ndahura NB (2012). Nutrition literacy status of adolescent students in Kampala district, Uganda, PhD thesis.Oslo and Akershus University College of Applied Sciences,Lillestrøm, Norway.

[30] Block LG, Grier SA, Childers TL, Davis B, Ebert JE, Kumanyika S, Laczniak RN, Machin JE, Motley CM, Peracchio L (2011). From nutrients to nurturance: A conceptual introduction to food well-being. J Public Policy Mark.

[31] Howard A, Brichta J. What’s to Eat? Improving Food Literacy in Canada.2013. [http://www.conferenceboard.ca/temp/d95c5003-64f9-43b3-bb90-0844b849460a/14-091_whatstoeat_cfic_rpt.pdf] Available from: [Accessed 1 Mar 2021].

[32] Thomas HM, Irwin JD (2011). Cook It Up! A community-based cooking program for at-risk youth: overview of a food literacy intervention. BMC Res Notes.

[33] Pendergast D, Garvis S, Kanasa H (2011). Insight from the public on home economics and formal food literacy. Fam Consum Sci Res J.

[34] Murimi MW (2013). Healthy literacy, nutrition education, and food literacy. J Nutr Educ Behav.

[35] Fordyce-Voorham S (2011). Identification of essential food skills for skill-based healthful eating programs in secondary schools. J Nutr Educ Behav.

[36] Topley, A. (2013). At the Table: A Case for Food Literacy Coordination. Greater Victoria Food Literacy Working Group, Victoria, BC [https://mail.google.com/mail/u/0/?ui=2&ik=14f4dc246b&view=att&th=13fab6fa0b29ac06&attid=0.2&disp=safe&zw.] [Accessed 2 Feb 2021].

[37] Watson WL, Chapman K, King L, Kelly B, Hughes C, Louie JCY, Crawford J, Gill TP (2013). How well do Australian shoppers understand energy terms on food labels?. Public Health Nutr.

[38] Desjardins, E. & Hailburton, K. (2013) Making something out of nothing: Food literacy among youth, young pregnant women and young parents who are at risk for poor health. Retrieved from the Ontario Society of Nutrition Professionals in Public Health. 2013. [http://www.osnpph.on.ca/resources/Food%20Literacy%20Study.LDCPOntario.Final.Dec2013.pdf ] Available from: [Accessed 1 Jan 2021].

[39] Guttersrud Ø, Dalane JØ, Pettersen S (2014). Improving measurement in nutrition literacy research using Rasch modelling: examining construct validity of stage-specific ‘critical nutrition literacy’scales. Public Health Nutr.

[40] Cullen T, Hatch J, Martin W, Higgins JW, Sheppard R (2015). Food Literacy: Definition and Framework for Action. Can J Diet Pract Res.

[41] Ronto R, Ball L, Pendergast D, Harris N (2016). Adolescents’ perspectives on food literacy and its impact on their dietary behaviours. Appetite.

[42] Wijayaratne SP, Reid M, Westberg K, Worsley A, Mavondo F (2018). Food literacy, healthy eating barriers and household diet. Eur J Mark.

[43] Poelman MP, Dijkstra SC, Sponselee H, Kamphuis CB, Battjes-Fries MC, Gillebaart M, Seidell JC (2018). Towards the measurement of food literacy with respect to healthy eating: the development and validation of the self perceived food literacy scale among an adult sample in the Netherlands. Int J Behav Nutr Phys Act.

[44] Begley A, Paynter E, Dhaliwal S (2018). Evaluation tool development for food literacy programs. Nutrients.

[45] Cullerton K, Vidgen HA, Gallegos D. A review of food literacy interventions targeting disadvantaged young people. Queensland University of Technology, School of Public Health. 2012. https://eprints.qut.edu.au/53753/. Accessed Aug 2022.

[46] Kolasa K, Lackey C (2006). The logic model as a framework for community program evaluations: the food literacy partners program. Fam Med.

[47] Sumner J (2015). Reading the world: Food literacy and the potential for food system transformation. Studies in the Education of Adults.

[48] Schnögl, S., Zehetgruber, R., Danninger, S., Setzwein, M., Wenk, R., Freudenberg, M., Müller, C., & Groeneveld, M. (2006). Savoury dishes for adult education and counselling. [www.foodliteracy.org.] Retrieved from [Accessed 10 Mar 2021].

[49] Vrhovnik L (2012). A pilot study for the development of a food skills survey tool. Thesis, Queen’s University, Kingston, Ontario, Canada.

[50] Bublitz M, Peracchio LA, Andreasen A, Kees J, Kidwell B, Miller E, Vallen B (2011). The quest for eating right: Advancing food well-being. J Res Consum.

[51] Rootman I, O’Neill M (2017). Key concepts in health promotion. Health Promotion in Canada: New Perspectives on Theory, Practice, Policy, and Research.

[52] Grové C, Laletas S: Promoting student wellbeing and mental health through social and emotional learning. In: Inclusive Education for the 21st Century. edn.: Routledge; 2020: 317–335.

[53] Krause C, Sommerhalder K, Beer-Borst S, Abel T (2018). Just a subtle difference? Findings from a systematic review on definitions of nutrition literacy and food literacy. Health Promot Int..

[54] Worsley A (2002). Nutrition knowledge and food consumption: can nutrition knowledge change food behaviour?. Asia Pac J Clin Nutr.

