# Supplementary material for: Development of a conceptual framework of food and nutrition literacy in children
Source: BMC Nutr. 2022 Aug 26;8:91. doi: 10.1186/s40795-022-00590-z (PMC9414122; doi:10.1186/s40795-022-00590-z)
Supplement: Supplementary file 1 — Additional file 1: Table S1. Question guides. [file 40795_2022_590_MOESM1_ESM.docx]

**Table S1: Question guides**

| **In-depth interview questions** | |
| --- | --- |
|  | In your opinion, what is the concept of food and nutrition literacy? |
|  | What components do you define for food and nutrition literacy? |
|  | Do you think there is a difference between food literacy and nutrition literacy? Which one is more important? |
|  | What are the components of food and nutrition literacy that elementary-school children should know? |
| **Focus group discussion questions** | |
|  | Have you ever considered the usefulness and harmfulness of the food you choose or eat? |
|  | Where do you get food and nutrition information? |
|  | How do you realize that your information is right or wrong? |
|  | What is your opinion about food advertisement in TV or other media? |
|  | What characteristics do you notice when buying food? |
|  | Can you make food yourself? |
|  | Who choose your snacks at school, you or others? |
