# Supplementary material for: Development of a conceptual framework of food and nutrition literacy in children
Source: BMC Nutr. 2022 Aug 26;8:91. doi: 10.1186/s40795-022-00590-z (PMC9414122; doi:10.1186/s40795-022-00590-z)
Supplement: Supplementary file 2 — Additional file 2: Table S2. Food/nutrition literacy definitions and conceptual frameworks with components of functional, interactive and critical food/nutrition literacy. [file 40795_2022_590_MOESM2_ESM.docx]

Table S2: Food/nutrition literacy definitions and conceptual frameworks with components of functional, interactive and critical food/nutrition literacy

| [Murimi (2013](#_ENREF_30)),USA [^1^](#_ENREF_1) | [Cullerton et al., (2012](#_ENREF_18)), Australia [^2^](#_ENREF_2) | Ndahura etal (2012), Uganda [^3^](#_ENREF_3) | [Thomas & Irwin (2011](#_ENREF_46)), London [^4^](#_ENREF_4) | [Pendergast et al (2011](#_ENREF_34)), Australia [^5^](#_ENREF_5) | [Block et al.(2011](#_ENREF_11)),USA [^6^](#_ENREF_6) | [Fordyce-Voorham (2011](#_ENREF_22)), Australia [^7^](#_ENREF_7) | [Zoellner et al.(2009](#_ENREF_53)), USA [^8^](#_ENREF_8) | Silk et al.(2008), USA [^9^](#_ENREF_9) | [Kolasa & Lackey (2006](#_ENREF_24)), USA [^10^](#_ENREF_10) | Blitstein & Evans (2006), USA[^11^](#_ENREF_11) | Schnoegl et al (2006), Germany^12^ | [Cardwell (2005](#_ENREF_15)),  USA [^13^](#_ENREF_13) | [Elrick et al.(2002](#_ENREF_20)), USA [^14^](#_ENREF_14) |  | **Studies** |
| --- | --- | --- | --- | --- | --- | --- | --- | --- | --- | --- | --- | --- | --- | --- | --- |
| adult | adolescents | adolescents | youth | adult | adult | adolescents | adult | adult | adult | adult | adult | adult | adult | **Target group** | |
|  |  |  |  |  |  |  |  |  |  |  |  |  |  | **Nutrition literacy definitions** | |
|  |  |  |  |  |  |  |  |  |  |  |  |  |  | **Food literacy definitions** | |
|  |  |  |  |  |  |  |  |  |  |  |  |  |  | **Based on Nutbeam’s model of health literacy** | |
|  |  |  |  |  |  |  |  |  |  |  |  |  |  | **Conceptual framework of food literacy** | |
|  |  |  |  |  |  |  |  |  |  |  |  |  |  | **Food/Nutrition literacy components** | |
|  |  |  |  |  |  |  |  |  |  |  |  |  |  | Nutrition knowledge |  |
|  |  |  |  |  |  |  |  |  |  |  |  |  |  | Lifestyle knowledge |  |
|  |  |  |  |  |  |  |  |  |  |  |  |  |  | Food safety knowledge | **Health-based knowledge** |
|  |  |  |  |  |  |  |  |  |  |  |  |  |  | Food knowledge and its preparation |  |
|  |  |  |  |  |  |  |  |  |  |  |  |  |  | Understanding food and nutrition information | **Understanding food & nutrition** |
|  |  |  |  |  |  |  |  |  |  |  |  |  |  | Access |  |
|  |  |  |  |  |  |  |  |  |  |  |  |  |  | Applying (healthy eating behaviours & food choices) | **Functional** |
|  |  |  |  |  |  |  |  |  |  |  |  |  |  | Interactive skills |  |
|  |  |  |  |  |  |  |  |  |  |  |  |  |  | Emotional skills | **Interactive** |
|  |  |  |  |  |  |  |  |  |  |  |  |  |  | Discussion Skills |  |
|  |  |  |  |  |  |  |  |  |  |  |  |  |  | Media literacy |  |
|  |  |  |  |  |  |  |  |  |  |  |  |  |  | Food label literacy | **Critical** |
|  |  |  |  |  |  |  |  |  |  |  |  |  |  | Decision-making and planning |  |
| 3 (2) | - | 9(4) | 2 (2) | 2 (2) | 1(1) | 1(1) | 1(1) | 1(1) | - | 1(1) | 4(3) | 1(1) | 1(1) | **Total components (domains)** | |

Table S2: Food/nutrition literacy definitions and conceptual frameworks with components of functional, interactive and critical food/nutrition literacy _(continued)_

| Truman et al (2019)  Canada [^15^](#_ENREF_15) | [Bublitz et al.(2019](#_ENREF_12)), USA [^16^](#_ENREF_16) | Begley et al (2018) Australia [^17^](#_ENREF_17) | Poelman et al (2018), Netherlands [^18^](#_ENREF_18) | Wijayaratne (2018) Australia [^19^](#_ENREF_19) | Ronto et al (2016), Australia [^20^](#_ENREF_20) | [Sumner (2015](#_ENREF_45))  Cnada [^21^](#_ENREF_21) | [Cullen et al. (2015](#_ENREF_17)) Cnada [^22^](#_ENREF_22) | [Sadegholvad et al. (2014](#_ENREF_41)),  Australia&Iran [^23^](#_ENREF_23) | Guttersrud et al.(2014),  Norway [^24^](#_ENREF_24) | [Vidgen & Gallegos (2014](#_ENREF_50)), Australia [^25^](#_ENREF_25) | Desjardins & Azevedo (2013), Canada ^26^ | Watson et al. (2013), Australia [^27^](#_ENREF_27) | Topley (2013),  Australia ^28^ | Howard & Brichta (2013), Canada [^29^](#_ENREF_29) | Slater (2013),  Canada [^30^](#_ENREF_30) |  | **Studies** | |
| --- | --- | --- | --- | --- | --- | --- | --- | --- | --- | --- | --- | --- | --- | --- | --- | --- | --- | --- |
| adult & youth | adult | adult | adult | adult | adolescents | adult | adult | adolescents | adult | adult & youth | adult | adult | adult | adult | children | **Target group** | | |
|  |  |  |  |  |  |  |  |  |  |  |  |  |  |  |  | **Nutrition literacy definitions** | | |
|  |  |  |  |  |  |  |  |  |  |  |  |  |  |  |  | **Food literacy definitions** | | |
|  |  |  |  |  |  |  |  |  |  |  |  |  |  |  |  | **Based on Nutbeam’s model of health literacy** | | |
|  |  |  |  |  |  |  |  |  |  |  |  |  |  |  |  | **Conceptual framework of food literacy** | | |
|  |  |  |  |  |  |  |  |  |  |  |  |  |  |  |  | **Food/Nutrition literacy components** | | |
|  |  |  |  |  |  |  |  |  |  |  |  |  |  |  |  | Nutrition knowledge | |  |
|  |  |  |  |  |  |  |  |  |  |  |  |  |  |  |  | Lifestyle knowledge | |  |
|  |  |  |  |  |  |  |  |  |  |  |  |  |  |  |  | Food safety knowledge | | **Health-based knowledge** |
|  |  |  |  |  |  |  |  |  |  |  |  |  |  |  |  | Food knowledge and its preparation | |  |
|  |  |  |  |  |  |  |  |  |  |  |  |  |  |  |  | Understanding food and nutrition information | | **Understanding food & nutrition** |
|  |  |  |  |  |  |  |  |  |  |  |  |  |  |  |  | Access | |  |
|  |  |  |  |  |  |  |  |  |  |  |  |  |  |  |  | Applying (healthy eating behaviours & food choices) | | **Functional** |
|  |  |  |  |  |  |  |  |  |  |  |  |  |  |  |  | Interactive skills | |  |
|  |  |  |  |  |  |  |  |  |  |  |  |  |  |  |  | Emotional skills | | **Interactive** |
|  |  |  |  |  |  |  |  |  |  |  |  |  |  |  |  | Discussion Skills | |  |
|  |  |  |  |  |  |  |  |  |  |  |  |  |  |  |  | Media literacy | |  |
|  |  |  |  |  |  |  |  |  |  |  |  |  |  |  |  | Food label literacy | | **Critical** |
|  |  |  |  |  |  |  |  |  |  |  |  |  |  |  |  | Decision-making and planning | |  |
| 6(3) | 2 (2) | 3(2) | 3 (2) | 3 (2) | 5(3) | - | 4(1) | 4(1) | 6(5) | 1(1) | 7(5) | 4(3) | 5(4) | 8(4) | 3(3) | **Total components (domains)** | | |

**References**

**1.** Murimi MW. Healthy literacy, nutrition education, and food literacy. *Journal of nutrition education and behavior.* 2013;45(3):195.

**2.** Cullerton K, Vidgen HA, Gallegos D. A review of food literacy interventions targeting disadvantaged young people.Queensland University of Technology, School of Public Health;. 2012.

**3.** Ndahura NB. Nutrition literacy status of adolescent students in Kampala district, Uganda, PhD thesis.Oslo and Akershus University College of Applied Sciences,Lillestrøm, Norway. 2012.

**4.** Thomas HM, Irwin JD. Cook It Up! A community-based cooking program for at-risk youth: overview of a food literacy intervention. *BMC Res Notes.* 2011;4:495.

**5.** Pendergast D, Garvis S, Kanasa H. Insight from the public on home economics and formal food literacy. *Family and Consumer Sciences Research Journal.* 2011;39(4):415-430.

**6.** Block LG, Grier SA, Childers TL, et al. From nutrients to nurturance: A conceptual introduction to food well-being. *Journal of Public Policy & Marketing.* 2011;30(1):5-13.

**7.** Fordyce-Voorham S. Identification of essential food skills for skill-based healthful eating programs in secondary schools. *J Nutr Educ Behav.* Mar-Apr 2011;43(2):116-122.

**8.** Zoellner J, Connell C, Bounds W, Crook L, Yadrick K. Nutrition literacy status and preferred nutrition communication channels among adults in the Lower Mississippi Delta. *Prev Chronic Dis.* Oct 2009;6(4):A128.

**9.** Silk KJ, Sherry J, Winn B, Keesecker N, Horodynski MA, Sayir A. Increasing nutrition literacy: testing the effectiveness of print, web site, and game modalities. *J Nutr Educ Behav.* Jan-Feb 2008;40(1):3-10.

**10.** Kolasa K, Lackey C. The logic model as a framework for community program evaluations: the food literacy partners program. *Fam Med.* Nov-Dec 2006;38(10):690-691.

**11.** Blitstein JL, Evans WD. Use of nutrition facts panels among adults who make household food purchasing decisions. *Journal of nutrition education and behavior.* 2006;38(6):360-364.

**12.** Schnoegl S., Zehetgruber R., Danninger S., Setzwein M., Wenk R., Freudenberg M., Mu¨ller C, Groeneveld M. (2006) Savoury Dishes for Adult Education and Counselling. Guidelines and Toolbox. In BEST Institut fu¨r berufsbezogene Weiterbildung und Personaltraining (ed).

**13.** Cardwell VB. Literacy: What Level for Food, Land, Natural Resources, and Environment? *Journal of natural resources and life sciences education.* 2005;34:112-117.

**14.** Elrick H, Samaras TT, Demas A. Missing links in the obesity epidemic. *Nutrition Research.* 2002;22(10):1101-1123.

**15.** Truman E, Elliott C. Barriers to food literacy: A conceptual model to explore factors inhibiting proficiency. *Journal of nutrition education and behavior.* 2019;51(1):107-111.

**16.** Bublitz MG, Hansen J, Peracchio LA, Tussler S. Hunger and Food Well-Being: Advancing Research and Practice. *Journal of Public Policy & Marketing.* 2019.

**17.** Begley A, Paynter E, Dhaliwal S. Evaluation tool development for food literacy programs. *Nutrients.* 2018;10(11):1617.

**18.** Poelman MP, Dijkstra SC, Sponselee H, et al. Towards the measurement of food literacy with respect to healthy eating: the development and validation of the self perceived food literacy scale among an adult sample in the Netherlands. *International Journal of Behavioral Nutrition and Physical Activity.* 2018;15(1):54.

**19.** Wijayaratne SP, Reid M, Westberg K, Worsley A, Mavondo F. Food literacy, healthy eating barriers and household diet. *European Journal of Marketing.* 2018;52(12):2449-2477.

**20.** Ronto R, Ball L, Pendergast D, Harris N. Adolescents' perspectives on food literacy and its impact on their dietary behaviours. *Appetite.* Dec 01 2016;107:549-557.

**21.** Sumner J. Reading the world: Food literacy and the potential for food system transformation. *Studies in the Education of Adults.* 2015;47(2):128-141.

**22.** Cullen T, Hatch J, Martin W, Higgins JW, Sheppard R. Food Literacy: Definition and Framework for Action. *Can J Diet Pract Res.* Sep 2015;76(3):140-145.

**23.** Sadegholvad S, Yeatman H, Omidvar N, Parrish A, Worsley A. What do school-leavers need to know about nutrition and food systems? Views of prominent food-related experts in iran. *Journal of Nutrition & Intermediary Metabolism.* 2014;1:11-12.

**24.** Guttersrud Ø, Dalane JØ, Pettersen S. Improving measurement in nutrition literacy research using Rasch modelling: examining construct validity of stage-specific ‘critical nutrition literacy’scales. *Public health nutrition.* 2014;17(4):877-883.

**25.** Vidgen HA, Gallegos D. Defining food literacy and its components. *Appetite.* May 2014;76:50-59.

**26.** Desjardins, E. & Hailburton, K. (2013) Making something out of nothing: Food literacy among youth, young pregnant women and young parents who are at risk for poor health. Retrieved from the Ontario Society of Nutrition Professionals in Public Health. 2013.Available from: <http://www.osnpph.on.ca/resources/Food%20Literacy%20Study.LDCPOntario.Final.Dec2013.pdf>

**27.** Watson WL, Chapman K, King L, et al. How well do Australian shoppers understand energy terms on food labels? *Public health nutrition.* 2013;16(3):409-417.

**28.** Topley, A. (2013). At the Table: A Case for Food Literacy Coordination. Greater Victoria Food Literacy Working Group, Victoria, BC https://mail.google.com/mail/u/0/?ui=2&ik=14f4dc246b&view=att&th=13fab6fa0b29ac06&attid=0.2&disp=safe&zw.

**29.** Howard, A., & Brichta, J. What's to Eat? Improving Food Literacy in Canada.2013. Available from: <http://www.conferenceboard.ca/temp/d95c5003-64f9-43b3-bb90-0844b849460a/14-091_whatstoeat_cfic_rpt.pdf>.

**30.** Slater J. Is cooking dead? The state of home economics food and nutrition education in a Canadian province. *International Journal of Consumer Studies.* 2013;37(6):617-624.
